# Editorial: Insights on cannabinoid translational science and medicine: the endocannabinoidome as a target for clinical practice

**DOI:** 10.3389/fnins.2024.1432892

**Published:** 2024-06-03

**Authors:** Luzia Sampaio, Raquel Maria Pereira Campos, Debra Karhson, Fabio Arturo Iannotti

**Affiliations:** ^1^Instituto de Biofísica Carlos Chagas Filho, Federal University of Rio de Janeiro, Rio de Janeiro, Brazil; ^2^Department of Psychology, University of New Orleans, New Orleans, LA, United States; ^3^Institute of Biomolecular Chemistry, National Research Council (CNR), Pozzuoli, Italy

**Keywords:** endocanabinoidome, *Cannabis sativa*, autism, medical cannabis, cannabinoid, epilepsy

In mammals and other vertebrates, the endocannabinoid system (ECS) is in a simplicistic manner formed by endocannabinoids, two cannabinoid receptors and large set of metabolic enzymes deputed to the synthesis and degradation of endocannabinoids. The ECS plays important physiological and homeostatic actions in the central nervous system (CNS), where it was discovered, but also in the majority (if not all) peripheral organs and tissue of the body especially those with endocrine, immune, gastrointestinal, reproductive functions. The identification of structure of the main phytocannabinoid present in *Cannabis sativa*, Δ^9^-tetrahydrocannabinol (THC) and cannabidiol (CBD), led to the discovery of the cannabinoid receptors first, and then also using synthetic cannabinoids, to the deciphering of the entire system (Cristino et al., 2020; Maccarrone et al., 2023).

The metabotropic cannabinoid receptor type 1 (CB1) and type 2 (CB2) are G protein coupled receptors (GPCR), with classically participation of Gi protein and inhibition of adenylate cyclase, but it can also be coupled to Gq/11 protein, leading to activation and modulation of activated kinases by mitogens (MAPKs) including extracellular signal-regulated kinases (ERK1/2) (Ye et al., 2019). Pharmacological studies have revealed the existence of other receptors capable of interacting with cannabinoids, such as the transient potential vanilloid receptor, type 1 (TRPV1) (Cristino et al., 2020).

Endocannabinoids are endogenous agonists of cannabinoid receptors, being classically derived from arachidonic acid and conjugated with an ethanolamine, dopamine or glycerol molecule. The first endocannabinoid discovered was *N*-arachidonoylethanolamine or anandamide (AEA), extracted from pig brains. This discovery allowed the identification of cannabimetic properties in another already known bioactive lipid, 2-arachidonoylglycerol (2-AG), which became the second endocannabinoid, both in the CNS and in peripheral tissues. In the following years, other endocannabinoids and congeners were isolated, however the biological activity and pharmacological proprieties of AEA and 2-AG remain the most studied (Fezza et al., 2014). The biosynthesis of different endocannabinoids is driven by an extensive number of enzymes, creating complex canonical and non-canonical pathways. These mechanisms are responsible for a main ECS characteristic, the on-demand production of endocannabinoids (Iannotti et al., 2016). Among the main enzymes of the ECS, phospholipase D selective for *N*-acyl-phosphatidylethanolamine (NAPE-PLD), fatty acid amide hydrolase (FAAH), diacylglycerol lipase (DAGL) and monoacylglycerol lipase (MAGL) are responsible for the synthesis and degradation of AEA and 2-AG, respectively (Maccarrone et al., 2023).

Recently, the new term endocannabinoidome, is started to be used to refer to the ECS in its expanded view, and thus encompass the new endocannabinoid and endocannabinoid-like mediators, signaling pathways, receptors and metabolic processes alternatives that can be modulated in health and disease. Therefore, the endocannabinoidome could be considered a valuable target in a pathological state. Considering all possible mechanisms for endocannabinoidome modulation, special attention is given to cannabinoids, substances capable of interacting with either receptors or other components of this system. Thus, scientific and medical efforts have sought to introduce synthetic and natural cannabinoids into clinical practice in order to improve patient's welfare (Pacher et al., 2019; Cristino et al., 2020).

*C. sativa* is one of the first plants in history to be used in medicine, its first use being described around 5,000 years ago, although it intense use in the medicine for centuries. Despite its medicinal potential the use of Cannabis was prohibited in several countries in the 20th century. Cannabinol (CBN) was the first phytocannabinoid to be isolated at the end of the 19th century. CBD was the second, although its chemical structure has not been correctly deciphered, while tetrahydrocannabinol was identified in 1942, but in a form of mixture of different tetrahydrocannabinols (Δ^8^-THC and Δ^9^-THC). Both CBD and THC, as other phytocannabinoids, are present in cannabis in acidic form, being decarboxylated when the plant is heated. Both the accurate structure, stereochemistry and synthesis of CBD and THC were complete elucidated by Professor Raphael Mechoulam in 1960 and in 1964 (Pertwee, 2006; Mechoulam et al., 2014). Mechoulam was a chemist, professor at the Faculty of Medicine of the Hebrew University of Jerusalem and his research opened door all over the word for a new era in the use of Cannabis in science and investigation of the potential in different pathological condition, including the fellowship with the Brazilian researcher professor Dr. Elisaldo Carlini to test CBD in epileptic patients (Cunha et al., 1980; Mechoulam, 2023). Unfortunately, we lost Dr. Mechoulam in February of 2023, during the time this editorial was opened to receive manuscript, and we are honored to receive one of the last works he was involved.

Currently, more than 520 active components extracted from Cannabis are known, including terpenes, flavonoids and more than 120 different phytocannabinoids, some of them acts as agonist and antagonists of CB1 and CB2 receptors (THC and Δ^9^-tetrahydrocannabivarin—THCV, respectively), and some have activity on the TRPV1 and TRPV2 receptor, such as CBD, THCV, cannabigerol (CBG), cannabigerovarin (CBGV), and cannabidivarin (CBDV) (Milay et al., 2020; Walsh et al., 2021). Phytocannabinoids can also exert important pharmacological effects by acting as ECS modulators, for instance, the acidic forms of phytocannabinoids can inhibit the AEA reuptake, while CBD which in addition to inhibiting AEA reuptake acts by inhibiting FAAH enzyme, in animal models, promoting an increase in intracellular levels of AEA (Di Marzo and Piscitelli, 2015). Although CBD has limited effects on cannabinoid receptors, it is known for anti-inflammatory and immunosuppressive action and can also antagonize some important effects of THC, such as anxiety, sedation and increased appetite (Russo, 2011). THC is associated with the modulation of signals that include pain, sedation, appetite and mood, and is also described as a bronchodilator, antioxidant with neuroprotective and anti-inflammatory properties. The action of THC on the CB1 receptor causes four symptoms characteristic of its psychotropic effects (inhibition of locomotor activity, hypothermia, catalepsy and antinociception), while its action on CB2 is correlated with the anti-inflammatory and relief action of pain (Keimpema et al., 2021).

The therapeutic potential of cannabis-derived products has been the subject of much research in recent years. These products have been used to treat a variety of conditions, including cancer, neuropathic pain, dementia, schizophrenia, epilepsy, and autism spectrum disorders (Cristino et al., 2020; Abrams et al., 2021; Aran et al., 2021; Campos et al., 2021; Devinsky et al., 2024). While the evidence supporting their use is growing, further research is needed to fully understand the pharmacological basis and cellular actions of these compounds in different disease contexts. This Research Topic aims to compile and critically evaluate the current scientific evidence on the use of Cannabinoid for medical purposes, providing a comprehensive overview of the field and highlighting areas for future research.

## Author contributions

LS: Writing – original draft, Writing – review & editing. RC: Writing – original draft, Writing – review & editing. DK: Writing – original draft, Writing – review & editing. FI: Writing – original draft, Writing – review & editing.

## References

[B1] Abrams D. I. Velasco G. Twelves C. Ganju R. K. Bar-Sela G. (2021). Cancer treatment: preclinical & clinical. J. Natl. Cancer Inst. Monogr. 2021, 107–113. 10.1093/jncimonographs/lgab010 34850894

[B2] Aran A. Harel M. Cassuto H. Polyansky L. Schnapp A. Wattad N.. (2021). Cannabinoid treatment for autism: a proof-of-concept randomized trial. Mol. Autism, 12:6. 10.1186/s13229-021-00420-2 33536055 PMC7860205

[B3] Campos R. M. P. Aguiar A. F. L. Paes-Colli Y. Trindade P. M. P. Ferreira B. K. de Melo Reis R. A.. (2021). Cannabinoid therapeutics in chronic neuropathic pain from animal research to human treatment. Front. Physiol. 12:785176. 10.3389/fphys.2021:785176 34916962 PMC8669747

[B4] Cristino L. Bisogno T. Di Marzo V. (2020). Cannabinoids and the expanded endocannabinoid system in neurological disorders. Nat. Rev. Neurol. 16, 9–29. 10.1038/s41582-019-0284-z 31831863

[B5] Cunha J. M. Carlini E. A. Pereira A. E. Ramos O. L. Pimentel C. Gagliardi R.. (1980). Chronic administration of cannabidiol to healthy volunteers and epileptic patients. Pharmacology 21, 175–185. 7413719 10.1159/000137430

[B6] Devinsky O. Jones N. A. Cunningham M. O. Jayasekera B. A. P. Whalley B. J. Devore S.. (2024). Cannabinoid treatments in epilepsy and seizure disorders. Physiol. Rev. 104, 591–649. 10.1152/physrev.00049.2021 37882730

[B7] Di Marzo V. Piscitelli F. (2015). The endocannabinoid system and its modulation by phytocannabinoids. Neurotherapeutics 12, 692–698. 10.1007/s13311-015-0374-6 26271952 PMC4604172

[B8] Fezza F. Bari M. Florio R. Talamonti E. Feole M. Maccarrone M.. (2014). Endocannabinoids, related compounds and their metabolic routes. Molecules 19, 17078–17106. 10.3390/molecules191117078 25347455 PMC6271436

[B9] Iannotti F. A. Di Marzo V. Petrosino S. (2016). Endocannabinoids and endocannabinoid-related mediators: targets, metabolism and role in neurological disorders. Prog. Lipid Res. 62, 107–128. 10.1016/j.plipres.2016.02.002 26965148

[B10] Keimpema E. Di Marzo V. Harkany T. (2021). Biological basis of cannabinoid medicines: mechanistic insights into cannabinoid signaling could improve therapeutic applications. Science 374, 1449–1450. 10.1126/science.abf6099 34914498

[B11] Maccarrone M. Di Marzo V. Gertsch J. Grether U. Howlett A. C. Hua T.. (2023). Goods and bads of the endocannabinoid system as a therapeutic target: lessons learned after 30 years. Pharmacol. Rev. 75, 885–958. 10.1124/pharmrev.122.000600 37164640 PMC10441647

[B12] Mechoulam R. (2023). A delightful trip along the pathway of cannabinoid and endocannabinoid chemistry and pharmacology. Annu. Rev. Pharmacol. Toxicol. 63, 1–13. 10.1146/annurev-pharmtox-051921-083709 35850522

[B13] Mechoulam R. Hanuš L. O. Pertwee R. Howlett A. C. (2014). Early phytocannabinoid chemistry to endocannabinoids and beyond. Nat. Rev. Neurosci. 15, 757–764. 10.1038/nrn3811 25315390

[B14] Milay L. Berman P. Shapira A. Guberman O. Meiri D. (2020). Metabolic profiling of cannabis secondary metabolites for evaluation of optimal postharvest storage conditions. Front. Plant Sci. 11:583605. 10.3389/fpls.2020.583605 33178249 PMC7593247

[B15] Pacher P. Kogan N. M. Mechoulam R. (2019). Beyond THC and endocannabinoids. Annu. Rev. Pharmacol. Toxicol. 60, 637–659. 10.1146/annurev-pharmtox-010818 31580774

[B16] Pertwee R. G. (2006). Cannabinoid pharmacology: the first 66 years. Br. J. Pharmacol. 147(Suppl 1), S163–S171. 10.1038/sj.bjp.0706406 16402100 PMC1760722

[B17] Russo E. B. (2011). Taming THC: potential cannabis synergy and phytocannabinoid-terpenoid entourage effects. Br. J. Pharmacol. 163, 1344–1364. 10.1111/bph.2011.163.issue-7 21749363 PMC3165946

[B18] Walsh K. B. McKinney A. E. Holmes A. E. (2021). Minor cannabinoids: biosynthesis, molecular pharmacology and potential therapeutic uses. Front. Pharmacol. 12:777804. 10.3389/fphar.2021.777804 34916950 PMC8669157

[B19] Ye L. Cao Z. Wang W. Zhou N. (2019). New insights in cannabinoid receptor structure and signaling. Curr. Mol. Pharmacol. 12, 239–248. 10.2174/1874467212666190215112036 30767756 PMC6864585

